# DNA methylation differences associated with social anxiety disorder and early life adversity

**DOI:** 10.1038/s41398-021-01225-w

**Published:** 2021-02-04

**Authors:** Ariane Wiegand, Benjamin Kreifelts, Matthias H. J. Munk, Nadja Geiselhart, Katia E. Ramadori, Julia L. MacIsaac, Andreas J. Fallgatter, Michael S. Kobor, Vanessa Nieratschker

**Affiliations:** 1 Department of Psychiatry and Psychotherapy, Eberhard Karls University of Tübingen, Tübingen, Germany; 2 Graduate Training Centre of Neuroscience, University of Tübingen, Tübingen, Germany; 3 Department of Biology, Technische Universität Darmstadt, Darmstadt, Germany; 4 Department of Medical Genetics, University of British Columbia, BC Children’s Hospital Research Institute, Vancouver, V5Z 4H4 BC Canada; 5 Werner Reichardt Centre for Integrative Neuroscience, Tübingen, Germany

**Keywords:** Clinical genetics, Psychiatric disorders

## Abstract

Social anxiety disorder (SAD) is a psychiatric disorder characterized by extensive fear in social situations. Multiple genetic and environmental factors are known to contribute to its pathogenesis. One of the main environmental risk factors is early life adversity (ELA). Evidence is emerging that epigenetic mechanisms such as DNA methylation might play an important role in the biological mechanisms underlying SAD and ELA. To investigate the relationship between ELA, DNA methylation, and SAD, we performed an epigenome-wide association study for SAD and ELA examining DNA from whole blood of a cohort of 143 individuals using DNA methylation arrays. We identified two differentially methylated regions (DMRs) associated with SAD located within the genes *SLC43A2* and *TNXB*. As this was the first epigenome-wide association study for SAD, it is worth noting that both genes have previously been associated with panic disorder. Further, we identified two DMRs associated with ELA within the *SLC17A3* promoter region and the *SIAH3* gene and several DMRs that were associated with the interaction of SAD and ELA. Of these, the regions within *C2CD2L* and *MRPL28* showed the largest difference in DNA methylation. Lastly, we found that two DMRs were associated with both the severity of social anxiety and ELA, however, neither of them was found to mediate the contribution of ELA to SAD later in life. Future studies are needed to replicate our findings in independent cohorts and to investigate the biological pathways underlying these effects.

## Introduction

Social anxiety disorder (SAD) is a psychiatric disorder characterized by intense fear in various social situations. Commonly, the fear of being the focus of attention and of negative evaluation play an essential role in the psychopathology and lead to severe impairments in daily life^1^. The etiology of social anxiety is influenced by genetic and environmental factors. One of the main environmental risk factors for SAD is early life adversity (ELA). Stressful experiences early in life, like childhood abuse and neglect, can lead to long-lasting behavioral and neurobiological changes ultimately contributing to an increased risk of SAD^2,3^. One mechanism that may explain how environmental factors can biologically contribute to psychopathological phenotypes is through epigenetics.

Epigenetic mechanisms can affect gene regulation without altering the DNA sequence. In human populations, the best-studied epigenetic modification is DNA methylation (DNAm), which refers to the covalent binding of a methyl group to the DNA. This occurs mostly, but not exclusively, on cytosines preceding a guanine, so called CpG sites^4^. DNAm is affected by genetic and environmental factors^5^. Since DNAm can be influenced by environmental stimuli and change over time, it might play an important role in adapting organisms to a changing environment, especially during vulnerable time periods including prenatal developmental stages and childhood^6^. Several studies have already linked ELA with long-lasting differences in DNAm levels^7^. Furthermore, differential DNAm patterns are also associated with SAD, amongst other neuropsychiatric disorders^8,9^. Hence, DNAm differences associated with ELA might be involved in the association of ELA with SAD later in life.

Previous research investigating differential DNAm associated with SAD has exclusively focused on candidate genes, such as the oxytocin receptor gene (*OXTR*)^10,11^. Only a few broader epigenome-wide association studies (EWAS) have been conducted for anxiety disorders and these have mainly investigated panic disorder^12,13^. Differential DNAm in association with ELA has been shown for several candidate genes, such as *SLC6A4*^14^, *FKBP5*^15^, and *NR3C1*^16^. In addition, studies investigating epigenome-wide DNAm in association with ELA, either in postmortem brain samples^17^ or peripheral tissues^18–21^, suggested differential DNAm in further genes such as *ALS2*^17^, *KITLG*^18^, *CYP2E1*^19^, and *SLC17A3*^21^ at various stages in life. Altogether, these findings indicate that long-lasting DNAm changes may be involved in the outcome of adverse experiences early in life.

In the current study we aimed to identify DNAm differences associated with the diagnosis of SAD and ELA on an epigenome-wide level and investigated the relationship between the effects of SAD and ELA on DNAm. To examine both divergent and convergent components of this interplay we first investigated, whether any DNAm changes associated with SAD were interacting with the experience of ELA. Secondly, we explored whether differential DNAm associated with the severity of both social anxiety and ELA were mediating the increased risk for SAD following ELA.

## Materials and methods

### Participants

In total, 143 participants of Caucasian descent between the ages of 18–50 years took part in the study. All participants were assessed using the Structured Clinical Interview for DSM-IV (SCID) and 66 participants were found to be presently suffering from SAD as a primary diagnosis. Supplementary Table S1 shows sample diagnostics and medication in more detail. The severity of social anxiety was evaluated using the Liebowitz Social Anxiety Scale (LSAS)^22^. ELA was assessed using the Childhood Trauma Questionnaire (CTQ) that measures five types of maltreatment: emotional and physical neglect and emotional, physical, and sexual abuse. Participants with at least a moderate score in one of the five categories were classified as participants with high levels of ELA^23,24^. Thus, four groups emerged: 1) control participants without SAD and low levels of ELA (Ce, *n* = 47), 2) control participants without SAD and high levels of ELA (CE, *n* = 30), 3) participants suffering from SAD with low levels of ELA (Se, *n* = 35), and 4) participants suffering from SAD with high levels of ELA (SE, *n* = 31). We also assessed self-reported measures of alcohol consumption, smoking behavior, and psychotropic medication. All participants gave written informed consent to the experimental procedure prior to inclusion in the study. The study was performed in accordance with the Declaration of Helsinki and approved by the University of Tübingen local ethics committee.

### DNA preparation and DNAm arrays

Blood samples were collected in EDTA tubes (9 ml Monovette^®^, Sarstedt, Sarstedt, GER), and stored at −80 °C. DNA was extracted using the QIAamp Blood Maxi Kit (Qiagen, Hilden, GER) according to manufacturer’s instructions. DNA was quantified using the Qubit^®^ 2.0 Fluorometer (Life Technologies, Carlsbad, CA) and stored at −20 °C.

Genomic DNA was bisulfite converted using the EZ DNA Methylation^TM^ Kit (Zymo Research, Irvine, CA) according to manufacturer’s instructions. DNAm was determined using the Infinium MethylationEPIC Kit (Illumina, San Diego, CA). To assess technical variation, one of the samples was run twice to generate a technical replicate. DNAm between those technical replicates had a Spearman correlation coefficient of rho >0.992 and a mean difference in methylation of 1.34% across all assessed CpG sites.

### Data quality control and normalization

Data processing and analyses were performed using the software R (Version 3.5.1)^25^. At first, the *getQC()* function from the *minfi* package was applied to the raw DNAm data to ensure high quality data. The median intensities for each sample were above the default threshold^26^. Further, all samples had <1% of probes with a detection *p*-value > 0.05.

For background and dye bias correction, the *preprocessNoob()* function from the *minfi* package was applied^27^. Additionally, we excluded 59 single nucleotide polymorphism (SNP) probes, 19,632 sex chromosome probes, 53,315 cross-reactive and polymorphic probes^28^, 4081 poorly performing probes (i.e., probes with a beadcount <3 in 5% of the samples and probes with a detection *p*-value > 0.05 in at least 1% of the samples), and 98,983 known invariant probes (inter-quantile range (IQR, 0.1−0.9) <0.02)^29^. Following these quality measures, 690,080 of the 866,150 original probes (79.7%) were left for further analyses.

To adjust for the bias originating from the two different bead types present on the BeadChip array, Beta-Mixture Quantile (BMIQ) normalization was performed^30^. To reduce batch effects of different runs, chips, and rows, the *ComBat()* function was applied^31^.

### Cell type estimates

To estimate the composition of different blood cell types within each sample, a reference-based deconvolution approach was applied. The *estimateCellCounts.wmln()* function from the *wateRmelon* package was used to estimate the relative proportions of monocytes, granulocytes, CD4^+^ and CD8^+^ T cells, B cells, and natural killer cells^32,33^. This method is based on the commonly used Houseman reference dataset for 450 K array data^34^. As actual blood cell counts for monocytes, granulocytes, and lymphocytes (no data for individual subtypes) were also available, the statistical estimates were compared to these cell counts (see Supplementary Fig. S1). DNAm values were adjusted for cell type composition using the statistical estimates in a regression-based approach^35^.

### Differential DNAm

On an epigenome-wide level, we extracted differentially methylated regions (DMRs) using the package *DMRcate*^36^. A linear model with the factors of interest SAD, ELA and their interaction and the covariates age and sex was fitted. We used a *p*-value cutoff of 0.05, a scaling factor of 2 and default parameters otherwise. For the main effects of SAD and ELA, all reported DMRs contained at least two CpG sites with an FDR-corrected *p*-value < 0.05 and a DNAm difference of >5%. For the interaction term, all reported DMRs contained at least three CpG sites with an FDR-corrected *p*-value < 0.05 and a DNAm range of >5% (i.e., at least two of the four groups had a DNAm difference >5%). Post-hoc pairwise comparisons were performed to investigate significant interaction effects.

For the top DMRs associated with SAD, Pearson correlation was assessed to investigate the association of the mean DNAm level of the identified sites with the total LSAS score, a measure of social anxiety. Likewise, for the top DMRs associated with ELA, Pearson correlation was used to test for an association of the mean DNAm level of the identified sites with the total and the subscale scores of the CTQ, an assessment of ELA (Bonferroni correction for multiple testing). All DMRs are reported using genomic coordinates from the human assembly GRCh37 (hg19) genome reference.

### Genetic influence

To investigate the genetic influence on DNAm in the identified DMRs, we screened the reported CpG sites of each region for methylation quantitative trait loci (mQTLs) using an mQTL database^37^. We only searched for genetic variants having an impact in “Middle Age” (i.e., the age group of our participants) using default parameters otherwise (i.e., database MatrixEQTL, trans distance 1,000,000 base pairs) and reported the number of different mQTLs for each DMR.

### Mediation analysis

To examine whether DNAm changes mediate increased social anxiety following ELA, a regression-based approach was performed using PROCESS (model 4)^38,39^. Two different linear models were fitted with one factor of interest, either total CTQ or total LSAS score, and the covariates age and sex. Using *DMRcate* with the same parameters as previously described, DMRs were identified for both models. Using a hypergeometric test, we examined whether the overlap of CpG sites predicted by both models was significant. For each DMR predicted by both total CTQ and total LSAS score, the mean DNAm was included as a mediating variable in a mediation model with the independent variable total CTQ score and the dependent variable total LSAS score and the covariates age and sex. The direct and indirect effects were tested using a percentile bootstrap estimation approach with 5000 samples.

## Results

### Study sample

Table 1 shows sample characteristics with respect to the four groups emerging from the factors SAD and ELA in more detail. While there was neither a significant group difference in age (*t*(141) = 0.05, *p* = 0.96) nor in sex (χ^2^ = 0.56, *p* = 0.45) with respect to SAD, significant differences in both age (*t*(85.8) = 2.60, *p* = 0.011) and sex (χ^2^ = 3.98, *p* = 0.046) emerged with respect to ELA. The total score of the CTQ was not significantly different with respect to sex (*t*(141) = 1.01, *p* = 0.32), but it correlated positively with age (*r* = 0.33, *p* < 0.001)^23,24^. For the total score of the LSAS there was no significant difference with respect to sex (*t*(141) = 1.66, *p* = 0.10) and no correlation with age (*r* = 0.02, *p* = 0.77)^22^. Furthermore, there was no difference with respect to the amount of alcohol consumed the month before study participation, neither with respect to SAD (*t*(124.7) = 0.90, *p* = 0.37), nor with respect to ELA (*t*(116.0) = 0.42, *p* = 0.68). Similarly, there was no significant difference in the number of cigarettes smoked during the month before study participation, neither for SAD (*t*(74.1) = 1.91, *p* = 0.06), nor for ELA (*t*(116.0) = 1.09, *p* = 0.28). Finally, there was a significant correlation between the total score of the LSAS and CTQ (*r* = 0.37, *p* < 0.001).

**Table 1:** Sample characteristics for the four groups emerging from the factors SAD and ELA.

	Ce	CE	Se	SE
*n*	47	30	35	31
Age [years]	25.0 (±4.3)	27.0 (±8.0)	23.9 (±4.5)	28.0 (±8.4)
Sex	31♀ 16♂	17♀ 13♂	29♀ 6♂	17♀ 14♂
LSAS	9.9 (±7.7)	20.1 (±17.7)	69.7 (±25.8)	73.3 (±28.6)
CTQ	33.5 (±6.3)	54.5 (±15.9)	37.1 (±7.3)	64.9 (±17.2)

Mean ± standard deviation. Ce control participants with low ELA level, CE control participants with high ELA level, Se participants suffering from SAD with low ELA levels, SE participants suffering from SAD with high ELA levels, LSAS Liebowitz Social Anxiety Scale, CTQ Childhood Trauma Questionnaire.

### Differential DNAm associated with SAD

**Fig. 1:**
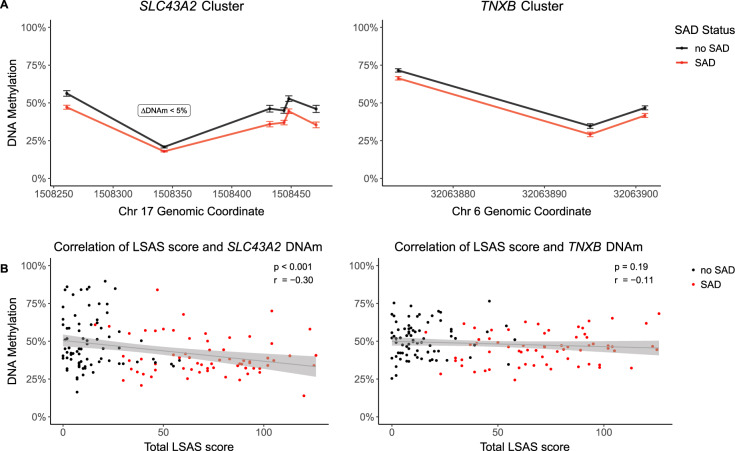
DMRs with respect to SAD. **A** Group mean DNAm ± standard error within the top two DMRs associated with SAD (*n* = 143). **B** Correlation of severity of social anxiety (LSAS score) with mean DNAm values of the *SLC43A2* (left) and the *TNXB* cluster (right). Regression line with 0.95 confidence interval is shown.

Investigating the DNAm data from all participants using DMRcate, eight differentially methylated CpG sites were identified which had a DNAm difference >5% and an FDR-corrected *p*-value < 0.05 with respect to SAD (see Supplementary Table S2). Five of these sites were in intron 5 of *SLC43A2* (p_FDR-corr_ < 5e-4) (DMR coordinates: chr17:1,508,261-1,509,247) and the remaining three sites were located in exon 4 of *TNXB* (p_FDR-corr_ < 3e-26) (DMR coordinates: chr6:32,062,885-32,065,702). Participants with SAD had decreased DNAm levels with a mean difference of 9.3% (*SLC43A2*) and 5.3% (*TNXB*) across the reported sites. Fig. 1A shows the section of each DMR containing these sites (whole DMRs in Supplementary Fig. S2).

Mean DNAm levels of the five CpG sites within *SLC43A2* showed a significant negative correlation with the total LSAS score (*r* = −0.30, *p* < 3e-4). In contrast, there was no significant correlation between *TNXB* DNAm levels and the total LSAS score (*r* = −0.11, *p* = 0.19) (Fig. 1B).

### Differential DNAm associated with ELA

**Fig. 2:**
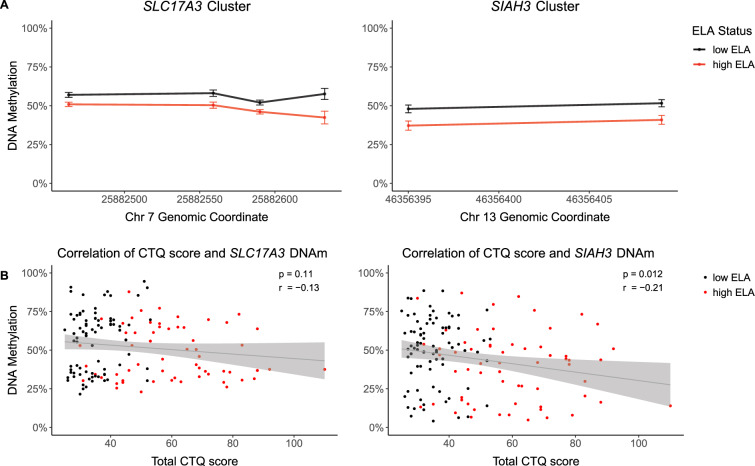
DMRs with respect to ELA. **A** Group mean DNAm ± standard error of the top two DMRs associated with ELA (*n* = 143). **B** Correlation of severity of ELA (total CTQ score) with mean DNAm values of the *SLC17A3* (left) and *SIAH3* cluster (right). Regression line with 0.95 confidence interval is shown.

Investigating the DNAm data from all participants, twelve CpG sites were identified to be differentially methylated with respect to ELA (see Supplementary Table S3). Four of these sites were located in the promoter region of *SLC17A3*, 8 kb upstream of exon 1 (*p*_FDR-corr_ < 0.035) (DMR coordinates: chr6:25,882,328-25,882,633). Another two sites were within exon 2 of the *SIAH3* gene (*p*_FDR-corr_ = 0.001) (DMR coordinates: chr13:46,355,841-46,356,409). In both regions, DNAm was lower in participants with high levels of ELA with a mean difference of 8.7% (*SLC17A3*) and 10.6% (*SIAH3*) across the reported sites. Figure 2A shows the section of each DMR containing these sites (whole DMRs in Supplementary Fig. S3).

DNAm levels in the four CpG sites within the *SLC17A3* promoter region showed no significant correlation with the total CTQ score (*r* = −0.13, *p* = 0.11) (Fig. 2B). When investigating CTQ subscales, a nominal significant, negative correlation was observed between *SLC17A3* DNAm and physical abuse (*r* = −0.17, *p* = 0.048). *SIAH3* DNAm levels showed a nominal significant, negative correlation with the total CTQ score (*r* = −0.21, *p* = 0.012) (Fig. 1B), and the subscale for emotional neglect (*r* = −0.19, *p* = 0.023), and a significant, negative correlation with emotional abuse (*r* = −0.24, *p* = 0.003) (all results in Supplementary Table S4).

### Differential DNAm associated with the interaction of SAD and ELA

**Fig. 3:**
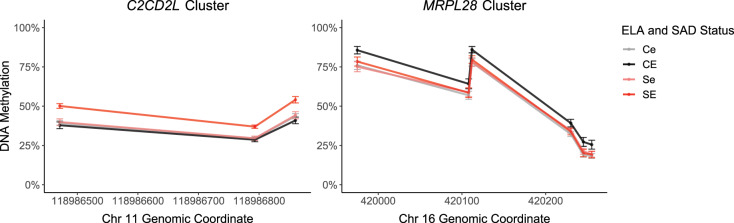
DMRs with respect to the interaction of SAD and ELA. Group mean DNAm ± standard error within the top two DMRs associated with the interaction of SAD and ELA (*n* = 143). Ce control participants with low ELA level, CE control participants with high ELA level, Se participants suffering from SAD with low ELA levels, SE participants suffering from SAD with high ELA levels.

With respect to the interaction of SAD and ELA, 107 CpG sites in 21 DMRs were identified (see Supplementary Table S5). The two DMRs with the largest DNAm range were located within the *C2CD2L* gene, spanning intron 13 and exon 14 (*p*_FDR-corr_ < 0.001) (DMR coordinates: chr11:118,986,471-118,987,153) and within the *MRPL28* gene, spanning exon 2 and partially spanning intron 1 and 2 (*p*_FDR-corr_ < 0.037) (DMR coordinates: chr16:419,800-420,326). Post-hoc *t*-tests showed that for the three CpG sites within *C2CD2L*, there was a significant difference in DNAm in participants suffering from SAD in association with ELA (*t*(64) = 3.78, *p* < 3.5e-4) but not in control participants (*t*(75) = 0.72, *p* = 0.47). Participants with SAD and high levels of ELA showed higher methylation in the *C2CD2L* region with a mean DNAm difference >9% compared to participants with SAD and low levels of ELA and the control participants. For the six CpG sites within *MRPL28*, there was a significant difference in DNAm in control participants in association with ELA (*t*(75) = 2.70, *p* = 0.009), but not in participants suffering from SAD (*t*(64) = 0.15, *p* = 0.88). Participants without SAD and high levels of ELA showed higher methylation with a mean difference in DNAm of >6% compared to control participants with low levels of ELA and participants suffering from SAD. Figure 3 shows these sites within the two DMRs (whole DMRs in Supplementary Fig. S4).

### Genetic influence on DMRs

Given that genotype also affects DNAm, we investigated the number of mQTLs for each DMR using an existing database^37^. The genetic influence on DNAm in the identified DMRs is diverse (Table 2). The highest number of mQTLs is reported for the *SLC17A3* cluster.

**Table 2:** Number of mQTLs influencing DNAm in each DMR.

DMR	# investigated CpG sites	# CpG sites in mQTL database	# mQTLs	Max. effect size
*SLC43A2* Cluster	5	2	62	0.08
*TNXB* Cluster	3	0	-	-
*SLC17A3* Cluster	4	2	821	0.26
*SIAH3* Cluster	2	0	-	-
*MRPL28* Cluster	6	4	384	0.22
*C2CD2L* Cluster	3	2	301	0.16

For each DMR, the number of reported CpG sites, the number of CpG sites available in the mQTL database, the number of associated mQTLs and their maximal effect sizes were listed.

### Mediation analysis

Investigating the DNAm data from all participants, 64 regions were identified where DNAm was significantly associated with the total CTQ score (*p*_FDR-corr_ < 0.05). Moreover, 122 regions were identified in association with the total LSAS score (*p*_FDR-corr_ < 0.05). Two of these regions were overlapping; one region included eight differentially methylated CpG sites (DMR coordinates: chr5:5,139,334-5,139,643) 0.8 kb upstream of *ADAMTS16*, and the other region included five overlapping CpG sites (DMR coordinates: chr18:74,961,138-74,961,494) located approximately 0.5 kb upstream of *GALR1*. Together, these 13 CpG sites represented a significant overlap (*p* < 6e-18).

To examine whether DNAm in these regions is mediating the increased risk for adult SAD after ELA, a mediation analysis based on the model shown in Supplementary Fig. S5 was performed. For *ADAMTS16*, the total CTQ score was a significant predictor of DNAm (*B* = 0.0004, SEM = 0.0002, *p* = 0.023) and *ADAMTS16* DNAm was a significant predictor of the total LSAS score (*B* = 146.935, SEM = 73.276, *p* = 0.047). While the direct effect was significant (*B* = 0.766, SEM = 0.168, 95% CI = 0.434, 1.099), the indirect effect of ELA on SAD mediated by DNAm was not significant (*B* = 0.065, SEM = 0.045, 95% CI = −0.009, 0.164).

For *GALR1*, the total CTQ score was a significant predictor of DNAm (*B* = 0.0002, SEM = 0.0001, *p* = 0.033) but *GALR1* DNAm was not a significant predictor of the total LSAS score (*B* = 169.517, SEM = 126.058, *p* = 0.18). While the direct effect of ELA on SAD was significant (*B* = 0.790, SEM = 0.169, 95% CI = 0.456, 1.124), the indirect effect mediated by methylation was not significant (*B* = 0.041, SEM = 0.043, 95% CI = −0.023, 0.144).

## Discussion

The present study represents a hypothesis-free EWAS for SAD, and, moreover, benefits from including ELA, one of the main environmental risk factors for SAD. Thus it afforded the investigation of the interrelations between SAD, ELA, and DNAm. We identified two DMRs associated with SAD, two DMRs associated with ELA, and several DMRs that exhibited differential DNAm associated with SAD interacting with ELA. An additional two DMRs where DNAm is associated with both the severity of ELA and of social anxiety were identified. However, there was no significant mediation effect of those epigenetic differences on the contribution of ELA to the severity of social anxiety later in life.

For SAD, we identified two DMRs, one in an intron of *SLC43A2* and one in the coding region of *TNXB*. *SLC43A2* belongs to the solute carrier (SLC) family and encodes the essential amino acid transporter LAT4. Changes in DNAm in response to psychotherapy have been reported in this gene, specifically an increase in DNAm in response to cognitive-behavioral therapy (CBT) and, hence, it was discussed to be potentially involved in the treatment response of CBT in panic disorder patients^40^. Remarkably, the reported site, cg22273830, is one of the four sites in our identified DMR. Assuming aberrant DNAm in anxiety patients reverts to levels of healthy controls in response to therapy, this is in line with the decreased methylation levels in individuals with SAD observed in our study. In this context, it is tempting to speculate that our results add evidence to the hypothesis that hypomethylation in this DMR might be involved in the pathophysiology of anxiety disorders. *TNXB* encodes tenascin-X, which plays an important role in connective tissue structure^41^. Several studies also suggest a genetic and epigenetic association between *TNXB* and psychiatric disorders such as schizophrenia^42,43^ or anorexia nervosa^44,45^ and, strikingly, hypomethylation of a CpG site in intron 6 of *TNXB* has already been associated with panic disorder in an EWAS^12^. As the present study is the first EWAS for SAD, it is worth noting that we identified two genes that may be involved in the underlying mechanisms of SAD, which have already been associated with panic disorder in previous EWAS, pointing towards a more general association with anxiety disorders.

Comparing participants with low and high ELA, we identified a region of the *SLC17A3* promoter and another region within *SIAH3* that exhibited differential DNAm. *SLC17A3* also belongs to the SLC family and encodes the sodium-phosphate transporter NPT4. A correlation analysis of our data revealed a nominal significant association of *SLC17A3* DNAm with physical abuse but not with emotional abuse and neglect, which might indicate a more specific effect of physical childhood abuse on *SLC17A3* DNAm. Interestingly, differential DNAm of the same CpG sites within the *SLC17A3* promoter has already been reported in a small cohort for childhood abuse victims^21^, although with an opposite direction of effect. Notably from our mQTL analysis, DNAm in this region was found to be potentially influenced by a high number of mQTLs. This genetic contribution might interfere with environmental effects if, by chance, groups are unbalanced for genotype. Therefore, especially in small cohorts, differential DNAm patterns in this region have to be interpreted with caution. The *SIAH3* gene encodes a member of the seven in absentia protein family and was suggested to be involved in the pathogenesis of neurodegenerative diseases^46^. When investigating different types of ELA, we specifically found a negative association of *SIAH3* DNAm with emotional abuse and a nominal significant association in the same direction with emotional neglect. In contrast, no association with physical or sexual childhood maltreatment was observed. Since *SIAH3* has not been previously associated with ELA or any stress-related disorders, it is important to validate this finding with future independent studies.

Several DMRs were associated with an interaction between SAD and ELA. Here, the largest DNAm differences were located within the genes *C2CD2L* and *MRPL28*. *C2CD2L* encodes the transmembrane protein 24 (TMEM24) which concentrates in the endoplasmic reticulum^47^. Of interest, *C2CD2L* has been associated with substance abuse on a genetic, expression, and DNAm level^48,49^. Furthermore, in adipose-derived stem cells, *C2CD2L* hypermethylation at one of the CpG sites in the identified DMR has been associated with low birth weight^50^, which in turn is associated with prenatal stress^51^. *MRPL28*, a mitochondrial ribosomal gene, has been found to be upregulated in the hypothalamus of mice that experienced chronic social defeat stress, a condition which can lead to anxiety- and depressive-like states^52^. In peripheral blood of infants, *MRPL28* hypermethylation at one of the CpG sites in the identified DMR has been associated with maternal asthma during pregnancy^53^, a maternal stressor which has consistently been associated with low birth weight^54^. Hence, hypermethylation in both genes might be associated with certain forms of prenatal stress. To date, an association with postnatal ELA has not been reported and it remains unclear how SAD might interact with potential effects. While for *C2CD2L* hypermethylation is only present in individuals suffering from SAD with high levels of ELA, *MRP28L* hypermethylation is only present in control participants with high ELA levels but not in any of the other groups. As individuals suffering from SAD with high levels of ELA showed increased *C2CD2L* DNAm compared to the remaining participants, there might be a cumulative effect of high levels of stress and anxiety during child- and adulthood on DNAm. In line with this, a previous study compared participants suffering from posttraumatic stress disorder (PTSD) with or without childhood abuse with control individuals without PTSD but trauma exposure. They reported a higher extent of DNAm differences in participants with PTSD and a history of childhood abuse compared to participants with PTSD but no history of childhood abuse^55^. For *MRPL28*, DNAm was found to be increased after high levels of ELA in control individuals but not in participants with SAD. One possible explanation might be a reversal of the long-lasting effect of ELA on DNAm in the presence of persisting clinically relevant levels of social anxiety. Additional studies are needed to investigate the underlying mechanisms of these effects. Nevertheless, the observed interaction effects highlight the importance of assessing ELA as an environmental factor influencing DNAm when investigating methylation changes in psychiatric disorders.

Lastly, since ELA is a risk factor for anxiety disorders such as SAD, we investigated whether DNAm differences may mediate this association. We identified two DMRs associated with both ELA and social anxiety, located upstream of the genes *ADAMTS16* and *GALR1*. *ADAMTS16* encodes a member of the ADAMTS (a disintegrin and metalloproteinase with thrombospondin motifs) protein family^56^. It has previously been associated with hypertension^57,58^ and, interestingly, hypertension has been linked with both the exposure to ELA and the diagnosis of early-onset SAD^59^. Perhaps the association of *ADAMTS16* DNAm with the severity of ELA and social anxiety in our data is related to chronic vegetative stress manifestation. Unfortunately, this hypothesis cannot be tested based on the present set of data since it did not include blood pressure measurements. *GALR1* encodes the galanin receptor type 1. Interestingly, several rodent studies have shown that the neuropeptide galanin, as well as its receptor subtypes, might play a role in depressive- and anxiety-like behavior^60,61^. Upon our mediation analysis, however, we did not find significant evidence that DNAm, neither in *ADAMTS16* nor in *GALR1*, statistically mediated the increased risk for SAD after ELA. One has to consider, though, that our study design may not have been ideally suited to investigate whether DNAm changes are involved in the manifestation of ELA in adult SAD. As the study design was primarily focused on interactions between ELA and SAD with the specific recruitment of four groups of participants with either high or low levels of ELA or social anxiety, the true degree of correlation between these two factors may have been underestimated in the present study with decreased sensitivity to detect potential mediators. To further investigate whether DNAm is a mediator of the manifestation of SAD in individuals with high levels of ELA, future studies should employ an unbiased recruitment strategy.

Other limitations of our study include potential confounding factors or the lack of potential explanatory data. Despite a well-characterized sample, further confounders could exist like the experience of stressful life events in adulthood or dietary differences. Additional explanatory data such as genotype data were not assessed in our study. Integrative approaches have shown that DNAm in the majority of variably methylated regions was best predicted by the combination of environmental factors and genotype information^62,63^. Although we were able to investigate an mQTL database^37^ and found many genetic variants affecting DNAm in some of the identified DMRs, especially within the *SLC17A3* promotor region, our study would have benefitted from combining genome-wide SNP data with epigenome-wide methylation data to provide further insights into the role of genetic variants on DNAm in the context of ELA and SAD.

Lastly, the biological interpretation of our findings is limited by several factors. Firstly, due to the lack of expression data, potential effects of observed DNAm differences on gene transcription remain unclarified. Secondly, due to the cell type and tissue specificity of epigenetic patterns, the DNAm levels assessed in whole blood, such as in the present study, and in the brain, where they can be assumed to be more directly linked to the pathophysiology of anxiety disorders, are not necessarily equivalent. Nevertheless, both ELA and SAD are associated with aberrant stress responsivity which can be argued to cause systemic effects. Another relevant limitation for this study is the relatively small sample size. Taking these limitations into consideration, however, we have demonstrated that several of our findings fit in well with previously reported results which in turn supports the reliability of our results. Still, future studies are needed to replicate our findings in independent, larger cohorts as a matter of course.

In summary, the present study is the first to examine differential DNAm associated with SAD on an epigenome-wide level and to study its relation to ELA. We identified several DMRs associated either with SAD, ELA, or their interaction. Interestingly, the two identified DMRs with respect to SAD have previously already been reported in association with panic disorder. Hence, they may be involved in psychophysiological mechanisms underlying anxiety disorders in a more general domain. Furthermore, we identified two DMRs associated with both the severity of ELA and social anxiety but neither of them could be proved to play a mediating role in the contribution of childhood adversities to social anxiety in adulthood. Translational studies investigating the pathophysiological relevance of the identified DMRs are required to understand their role in disease vulnerability or pathogenesis.

## Supplementary information

Supplementary Information
