# Supplementary material for: DNA methylation differences associated with social anxiety disorder and early life adversity
Source: Transl Psychiatry. 2021 Feb 4;11:104. doi: 10.1038/s41398-021-01225-w (PMC7862482; doi:10.1038/s41398-021-01225-w)
Supplement: Supplementary file 1 — Supplementary Information [file 41398_2021_1225_MOESM1_ESM.docx]

## Supplementary Information

**Supplementary Table S1. Clinical sample description for the four groups emerging from the factors SAD and ELA.**

|  | Ce | CE | Se | SE |
| --- | --- | --- | --- | --- |
| Total participants | 47 | 30 | 35 | 31 |
| Diagnosis |  |  |  |  |
| Current depression  (mild-moderate) | 0 | 0 | 3 | 4 |
| Dysthymia | 1 | 0 | 5 | 4 |
| Alcohol abuse | 1 | 1 | 0 | 1 |
| Drug abuse | 0 | 0 | 0 | 0 |
| Panic disorder | 0 | 0 | 6 | 11 |
| Agoraphobia | 0 | 0 | 5 | 6 |
| Specific phobia | 2 | 5 | 6 | 5 |
| Obsessive–compulsive disorder | 0 | 1 | 0 | 2 |
| Posttraumatic stress disorder | 0 | 0 | 0 | 0 |
| Generalized anxiety disorder | 1 | 0 | 6 | 3 |
| Eating disorder | 0 | 0 | 1 | 0 |
| Medication |  |  |  |  |
| Benzodiazepines | 0 | 0 | 2 | 1 |
| Antidepressants | 0 | 0 | 2* | 3** |

Ce: control participants with low ELA level, CE: control participants with high ELA level, Se: participants suffering from SAD with low ELA levels, SE: participants suffering from SAD with high ELA levels

* Escitalopram, Opipramol

** Escitalopram, Citalopram, Fluoxetin

**Supplementary Figure S1. Comparison of statistical cell type estimates with blood count data.** Correlation of reference-based cell type estimates with actual blood cell counts.

**Supplementary Table S2. DMRs in association with SAD.** 8 CpG sites form 2 DMRs showed a DNAm difference > 5% and an FDR-corrected p-value < 0.05.

| CpG site | FDR | Gene | Position | DMR coord. | DNAm Diff. |
| --- | --- | --- | --- | --- | --- |
| cg09329516 | 0.0005 | *SLC43A2* | 1508261 | chr17:1508261-1509247 | -0.0915 |
| cg26423139 | 0.0003 | *SLC43A2* | 1508432 | chr17:1508261-1509247 | -0.1026 |
| cg11076954 | 0.0003 | *SLC43A2* | 1508444 | chr17:1508261-1509247 | -0.0800 |
| cg22297055 | 0.0002 | *SLC43A2* | 1508448 | chr17:1508261-1509247 | -0.0833 |
| cg22273830 | 0.0002 | *SLC43A2* | 1508471 | chr17:1508261-1509247 | -0.1059 |
| cg10365886 | 2.99E-26 | *TNXB* | 32063874 | chr6:32062885-32065702 | -0.0522 |
| cg14188106 | 1.65E-26 | *TNXB* | 32063895 | chr6:32062885-32065702 | -0.0550 |
| cg07524919 | 1.49E-26 | *TNXB* | 32063901 | chr6:32062885-32065702 | -0.0507 |


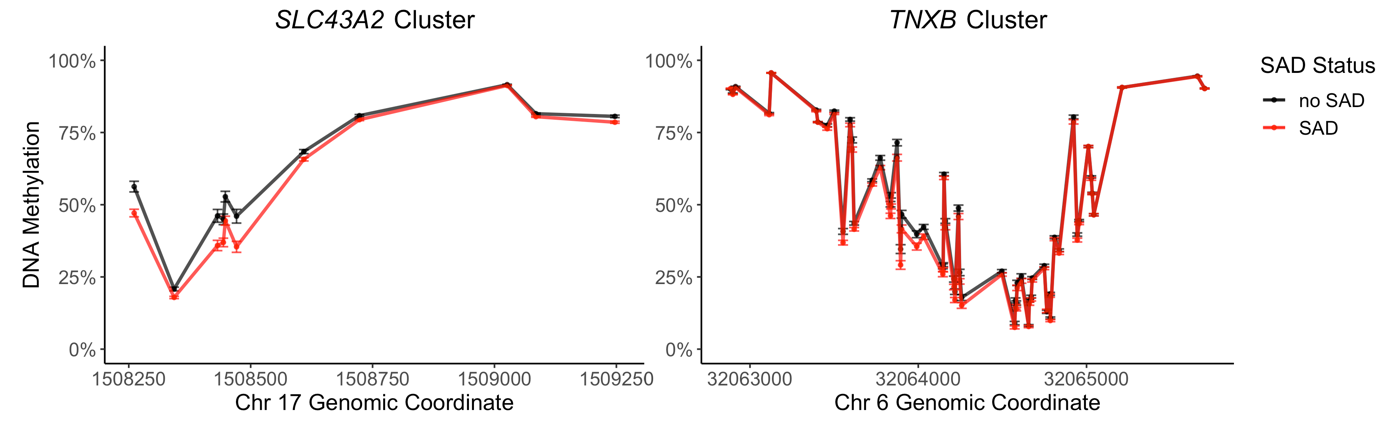


**Supplementary Figure S2. DMRs with respect to SAD.** Group mean DNAm ± standard error of the top two DMRs associated with SAD (n=143).

**Supplementary Table S3. DMRs in association with ELA.** 12 CpG sites from 8 DMRs showed a DNAm difference > 5% and an FDR-corrected p-value < 0.05.

| CpG site | FDR | Gene | Position | DMR coord. | DNAm Diff. |
| --- | --- | --- | --- | --- | --- |
| cg24065597 | 0.0277 | *SLC17A3* | 25882633 | chr6:25882328-25882633 | -0.1521 |
| cg01615050 | 0.0314 | *SLC17A3* | 25882559 | chr6:25882328-25882633 | -0.0773 |
| cg03264133 | 0.0348 | *SLC17A3* | 25882463 | chr6:25882328-25882633 | -0.0611 |
| cg03517284 | 0.0230 | *SLC17A3* | 25882590 | chr6:25882328-25882633 | -0.0594 |
| cg04924408 | 0.0010 | *SIAH3* | 46356395 | chr13:46355841-46356409 | -0.1077 |
| cg03997626 | 0.0010 | *SIAH3* | 46356409 | chr13:46355841-46356409 | -0.1073 |
| cg23302570 | 0.0383 | *NXPH2* | 139538601 | chr2:139538222-139538601 | 0.0762 |
| cg22511951 | 0.0084 | *-* | 7750480 | chr4:7750448-7750489 | -0.0681 |
| cg15848685 | 0.0377 | *VARS2* | 30882641 | chr6:30882641-30883001 | -0.0646 |
| cg26913058 | 0.0011 | *MRPL28* | 419975 | chr16:419800-420755 | 0.0635 |
| cg01178624 | 0.0022 | *KCNK7* | 65360327 | chr11:65360123-65360620 | -0.0584 |
| cg08185624 | 0.0265 | *LINC01179; LOC101928131* | 166662150 | chr4:166662150-166662284 | -0.0523 |


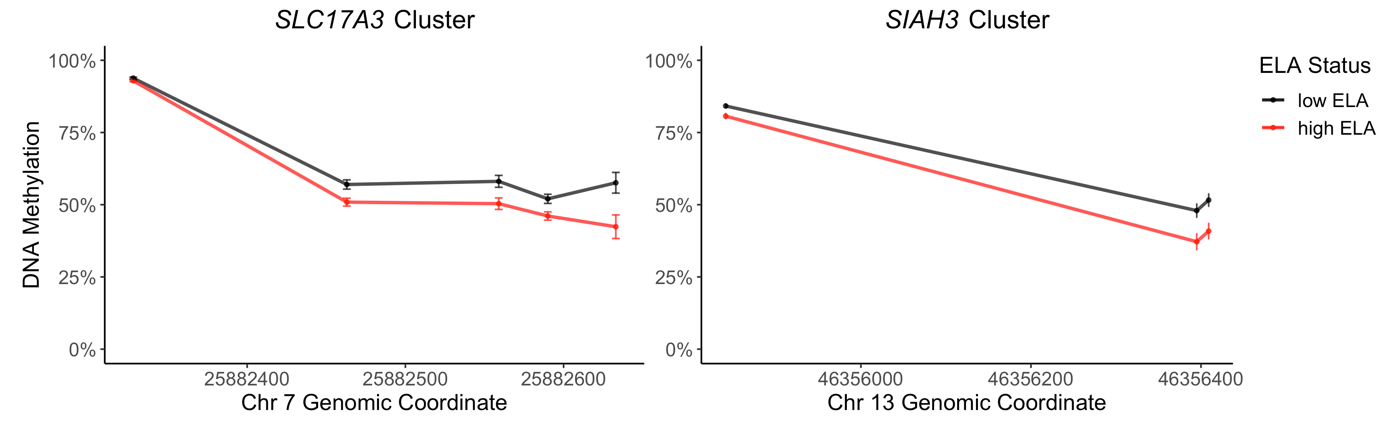


**Supplementary Figure S3. DMRs with respect to ELA.** Group mean DNAm ± standard error of the top two DMRs associated with ELA (n=143).

**Supplementary Table S4. Pearson correlation of mean DNAm levels in the identified DMRs and CTQ scales.**

|  | ***SLC17A3*** | | ***SIAH3*** | |
| --- | --- | --- | --- | --- |
|  | r | p | r | p |
| Emotional abuse | -0.12 | 0.144 | **-0.24*** | **0.003*** |
| Physical abuse | **-0.17** | **0.048** | -0.14 | 0.102 |
| Sexual abuse | 0.02 | 0.846 | -0.01 | 0.904 |
| Emotional neglect | -0.13 | 0.125 | **-0.19** | **0.023** |
| Physical neglect | -0.15 | 0.076 | -0.16 | 0.057 |
| Total CTQ score | -0.13 | 0.112 | **-0.21** | **0.012** |

Results with a nominal p-value < 0.05 printed in bold; significant results (i.e. after correction for multiple testing) denoted by asterisk. CTQ: Childhood Trauma Questionnaire.

**Supplementary Table S5. DMRs in association with the interaction of SAD and ELA.** 107 CpG sites from 21 DMRs with at least 3 CpG sites showing a DNAm difference > 5% between any two groups and an FDR-corrected p-value < 0.05.

| CpG site | FDR | Gene | Position | DMR coord. | Ce DNAm | CE DNAm | Se DNAm | SE DNAm |
| --- | --- | --- | --- | --- | --- | --- | --- | --- |
| cg18876294 | 0.0006 | *C2CD2L* | 118986471 | chr11:118986471-118987153 | 0.3927 | 0.3786 | 0.3992 | 0.5012 |
| cg15212210 | 0.0008 | *C2CD2L* | 118986793 | chr11:118986471-118987153 | 0.2940 | 0.2863 | 0.2956 | 0.3696 |
| cg22259797 | 0.0010 | *C2CD2L* | 118986860 | chr11:118986471-118987153 | 0.4361 | 0.4094 | 0.4425 | 0.5411 |
| cg26913058 | 0.0345 | *MRPL28* | 419975 | chr16:419800-420326 | 0.7599 | 0.8565 | 0.7513 | 0.7842 |
| cg00101154 | 0.0322 | *MRPL28* | 420108 | chr16:419800-420326 | 0.5694 | 0.6423 | 0.5880 | 0.5856 |
| cg12437481 | 0.0323 | *MRPL28* | 420112 | chr16:419800-420326 | 0.7787 | 0.8599 | 0.7805 | 0.7968 |
| cg08923669 | 0.0352 | *MRPL28* | 420230 | chr16:419800-420326 | 0.3224 | 0.3920 | 0.3452 | 0.3384 |
| cg17422692 | 0.0360 | *MRPL28* | 420245 | chr16:419800-420326 | 0.1914 | 0.2726 | 0.2129 | 0.2013 |
| cg08296601 | 0.0367 | *MRPL28* | 420255 | chr16:419800-420326 | 0.1832 | 0.2551 | 0.1940 | 0.1922 |
| cg25910261 | <0.0001 | *PTPRN2* | 157405965 | chr7:157405965-157406737 | 0.2343 | 0.2080 | 0.1652 | 0.2495 |
| cg22970003 | <0.0001 | *PTPRN2* | 157406032 | chr7:157405965-157406737 | 0.2154 | 0.1924 | 0.1637 | 0.2275 |
| cg23299919 | <0.0001 | *PTPRN2* | 157406096 | chr7:157405965-157406737 | 0.4054 | 0.3598 | 0.3213 | 0.4230 |
| cg14091258 | <0.0001 | *PTPRN2* | 157406607 | chr7:157405965-157406737 | 0.6096 | 0.5819 | 0.5683 | 0.6207 |
| cg09419670 | 0.0376 | *PSMD5; LOC253039* | 123605666 | chr9:123605666-123606675 | 0.2826 | 0.3376 | 0.3297 | 0.2325 |
| cg03499324 | 0.0001 | *PSMD5-AS1; PSMD5* | 123606119 | chr9:123605666-123606675 | 0.8261 | 0.8589 | 0.8542 | 0.8037 |
| cg14255062 | 0.0013 | *PSMD5* | 123606675 | chr9:123605666-123606675 | 0.8048 | 0.8342 | 0.8260 | 0.7839 |
| cg23978242 | 0.0002 | *DIP2C* | 530635 | chr10:530635-531914 | 0.5966 | 0.5587 | 0.5398 | 0.6032 |
| cg27421267 | <0.0001 | *DIP2C* | 530836 | chr10:530635-531914 | 0.6647 | 0.6141 | 0.5843 | 0.6318 |
| cg00219169 | <0.0001 | *DIP2C* | 530951 | chr10:530635-531914 | 0.6315 | 0.5987 | 0.5766 | 0.6151 |
| cg09955645 | <0.0001 | *DIP2C* | 531098 | chr10:530635-531914 | 0.6275 | 0.5970 | 0.5772 | 0.6112 |
| cg15105011 | 0.0213 | *TMEM175* | 940614 | chr4:940614-941054 | 0.6611 | 0.6348 | 0.6107 | 0.6817 |
| cg23939001 | 0.0209 | *TMEM175* | 940644 | chr4:940614-941054 | 0.6110 | 0.5943 | 0.5749 | 0.6360 |
| cg08222618 | 0.0176 | *TMEM175* | 941054 | chr4:940614-941054 | 0.4871 | 0.4705 | 0.4585 | 0.5211 |
| cg09075844 | 0.0001 | *SLC6A18* | 1245902 | chr5:1245669-1246795 | 0.5855 | 0.5704 | 0.5247 | 0.5965 |
| cg27432784 | 0.0001 | *SLC6A18* | 1246254 | chr5:1245669-1246795 | 0.2954 | 0.2789 | 0.2454 | 0.3074 |
| cg02287371 | 0.0001 | *SLC6A18* | 1246274 | chr5:1245669-1246795 | 0.2406 | 0.2135 | 0.1844 | 0.2390 |
| cg14003022 | <0.0001 |  | 3043019 | chr4:3042635-3043752 | 0.5860 | 0.5896 | 0.5331 | 0.5917 |
| cg02566259 | <0.0001 |  | 3043199 | chr4:3042635-3043752 | 0.3725 | 0.3365 | 0.2792 | 0.3724 |
| cg01807241 | <0.0001 |  | 3043527 | chr4:3042635-3043752 | 0.2791 | 0.2431 | 0.1994 | 0.2794 |
| cg06421590 | <0.0001 |  | 3043752 | chr4:3042635-3043752 | 0.2988 | 0.2653 | 0.2243 | 0.2972 |
| cg17759252 | 0.0495 | *CRTC1* | 18888081 | chr19:18888081-18889003 | 0.7932 | 0.7345 | 0.7108 | 0.7796 |
| cg15190383 | 0.0073 | *CRTC1* | 18888799 | chr19:18888081-18889003 | 0.6158 | 0.5722 | 0.5632 | 0.6151 |
| cg05365033 | 0.0133 | *CRTC1* | 18889003 | chr19:18888081-18889003 | 0.6761 | 0.6238 | 0.6171 | 0.6758 |
| cg01286685 | <0.0001 | *RNF39* | 30039025 | chr6:30038004-30039801 | 0.4898 | 0.4772 | 0.4647 | 0.5242 |
| cg00947782 | <0.0001 | *RNF39* | 30039142 | chr6:30038004-30039801 | 0.0987 | 0.0947 | 0.0752 | 0.1354 |
| cg03343571 | <0.0001 | *RNF39* | 30039175 | chr6:30038004-30039801 | 0.1719 | 0.1690 | 0.1455 | 0.2296 |
| cg13185413 | <0.0001 | *RNF39* | 30039202 | chr6:30038004-30039801 | 0.0961 | 0.0838 | 0.0796 | 0.1338 |
| cg06249604 | <0.0001 | *RNF39* | 30039206 | chr6:30038004-30039801 | 0.1321 | 0.1203 | 0.1025 | 0.1919 |
| cg20249327 | <0.0001 | *RNF39* | 30039374 | chr6:30038004-30039801 | 0.1269 | 0.1335 | 0.1091 | 0.1691 |
| cg15877520 | <0.0001 | *RNF39* | 30039376 | chr6:30038004-30039801 | 0.0720 | 0.0656 | 0.0531 | 0.1045 |
| cg09279736 | <0.0001 | *RNF39* | 30039403 | chr6:30038004-30039801 | 0.2038 | 0.2068 | 0.1695 | 0.2503 |
| cg07382347 | <0.0001 | *RNF39* | 30039408 | chr6:30038004-30039801 | 0.0888 | 0.0842 | 0.0648 | 0.1337 |
| cg13401893 | <0.0001 | *RNF39* | 30039432 | chr6:30038004-30039801 | 0.1919 | 0.1831 | 0.1636 | 0.2622 |
| cg12633154 | <0.0001 | *RNF39* | 30039435 | chr6:30038004-30039801 | 0.1824 | 0.1726 | 0.1518 | 0.2567 |
| cg16078649 | <0.0001 | *RNF39* | 30039466 | chr6:30038004-30039801 | 0.1226 | 0.1216 | 0.1037 | 0.1706 |
| cg10930308 | <0.0001 | *RNF39* | 30039476 | chr6:30038004-30039801 | 0.1402 | 0.1270 | 0.1083 | 0.1837 |
| cg24103044 | <0.0001 | *RNF39* | 30039801 | chr6:30038004-30039801 | 0.4330 | 0.4207 | 0.4060 | 0.4592 |
| cg27387193 | 0.0377 | *TNXB* | 32064032 | chr6:32064032-32065211 | 0.4193 | 0.4293 | 0.3676 | 0.4136 |
| cg17662683 | 0.0132 | *TNXB* | 32064146 | chr6:32064032-32065211 | 0.2837 | 0.2912 | 0.2382 | 0.2841 |
| cg01992382 | 0.0070 | *TNXB* | 32064212 | chr6:32064032-32065211 | 0.2392 | 0.2475 | 0.1933 | 0.2397 |
| cg16834823 | 0.0066 | *TNXB* | 32064218 | chr6:32064032-32065211 | 0.1920 | 0.2100 | 0.1510 | 0.1915 |
| cg00525277 | 0.0054 | *TNXB* | 32064239 | chr6:32064032-32065211 | 0.4849 | 0.4939 | 0.4363 | 0.4849 |
| cg10890302 | 0.0051 | *TNXB* | 32064246 | chr6:32064032-32065211 | 0.2587 | 0.2759 | 0.2132 | 0.2587 |
| cg10923662 | 0.0047 | *TNXB* | 32064258 | chr6:32064032-32065211 | 0.1757 | 0.1827 | 0.1299 | 0.1754 |
| cg01664727 | 0.0164 | *RUNX1* | 36258423 | chr21:36258423-36259797 | 0.2112 | 0.1668 | 0.1943 | 0.2256 |
| cg03142697 | 0.0117 | *RUNX1* | 36258497 | chr21:36258423-36259797 | 0.3414 | 0.2788 | 0.3099 | 0.3451 |
| cg26974661 | 0.0072 | *RUNX1* | 36258596 | chr21:36258423-36259797 | 0.3321 | 0.2817 | 0.3045 | 0.3375 |
| cg12477880 | 0.0076 | *RUNX1* | 36259241 | chr21:36258423-36259797 | 0.3575 | 0.3068 | 0.3417 | 0.3608 |
| cg12083928 | 0.0158 | *RUNX1* | 36259623 | chr21:36258423-36259797 | 0.2503 | 0.2032 | 0.2341 | 0.2580 |
| cg20718640 | 0.0063 | *PDXK* | 45160784 | chr21:45160581-45161603 | 0.8316 | 0.8666 | 0.8831 | 0.8202 |
| cg14583312 | 0.0098 | *PDXK* | 45161273 | chr21:45160581-45161603 | 0.2555 | 0.2950 | 0.3173 | 0.2707 |
| cg13159023 | 0.0102 | *PDXK* | 45161285 | chr21:45160581-45161603 | 0.2612 | 0.2981 | 0.3251 | 0.2795 |
| cg04699312 | 0.0107 | *PDXK* | 45161297 | chr21:45160581-45161603 | 0.2158 | 0.2457 | 0.2671 | 0.2303 |
| cg19815989 | 0.0134 | *PDXK* | 45161359 | chr21:45160581-45161603 | 0.5977 | 0.6607 | 0.6604 | 0.6207 |
| cg02873954 | 0.0163 | *PDXK* | 45161415 | chr21:45160581-45161603 | 0.5771 | 0.6393 | 0.6444 | 0.5851 |
| cg26079664 | 0.0069 | *GRM2* | 51740956 | chr3:51740622-51741473 | 0.2950 | 0.2821 | 0.2656 | 0.3165 |
| cg12934382 | 0.0039 | *GRM2* | 51741135 | chr3:51740622-51741473 | 0.2631 | 0.2470 | 0.2260 | 0.2892 |
| cg21213853 | 0.0020 | *GRM2* | 51741473 | chr3:51740622-51741473 | 0.6025 | 0.5866 | 0.5724 | 0.6358 |
| cg24820672 | <0.0001 |  | 112984728 | chr13:112984478-112986978 | 0.6465 | 0.5958 | 0.5487 | 0.6331 |
| cg27260867 | <0.0001 |  | 112984840 | chr13:112984478-112986978 | 0.6143 | 0.5830 | 0.5612 | 0.6085 |
| cg19783563 | <0.0001 | *LINC01044* | 112985611 | chr13:112984478-112986978 | 0.4374 | 0.4107 | 0.3933 | 0.4467 |
| cg13988338 | <0.0001 |  | 112986154 | chr13:112984478-112986978 | 0.2939 | 0.2596 | 0.2464 | 0.3006 |
| cg03486986 | <0.0001 |  | 112986285 | chr13:112984478-112986978 | 0.2568 | 0.2359 | 0.2035 | 0.2815 |
| cg16288318 | 0.0018 |  | 112986927 | chr13:112984478-112986978 | 0.6102 | 0.5790 | 0.5455 | 0.5859 |
| cg22762992 | 0.0174 |  | 118616377 | chr2:118616155-118616576 | 0.7267 | 0.7505 | 0.7892 | 0.7381 |
| cg06940110 | 0.0157 |  | 118616430 | chr2:118616155-118616576 | 0.4984 | 0.5310 | 0.5563 | 0.5064 |
| cg24213669 | 0.0125 |  | 118616576 | chr2:118616155-118616576 | 0.5234 | 0.5559 | 0.5802 | 0.5297 |
| cg05524354 | 0.0096 | *PTPRE* | 129797760 | chr10:129797760-129797840 | 0.5040 | 0.5227 | 0.5522 | 0.4830 |
| cg24824686 | 0.0096 | *PTPRE* | 129797777 | chr10:129797760-129797840 | 0.6597 | 0.6802 | 0.6980 | 0.6368 |
| cg11659652 | 0.0096 | *PTPRE* | 129797840 | chr10:129797760-129797840 | 0.7341 | 0.7526 | 0.7729 | 0.7131 |
| cg27261050 | <0.0001 | *PIWIL1* | 130821607 | chr12:130821453-130824831 | 0.6259 | 0.6197 | 0.5893 | 0.6525 |
| cg24229701 | <0.0001 | *PIWIL1* | 130821962 | chr12:130821453-130824831 | 0.6568 | 0.6333 | 0.6131 | 0.6653 |
| cg18319102 | 0.0005 | *PIWIL1* | 130822256 | chr12:130821453-130824831 | 0.3730 | 0.3381 | 0.3314 | 0.3962 |
| cg13861644 | 0.0007 | *PIWIL1* | 130822286 | chr12:130821453-130824831 | 0.7358 | 0.6802 | 0.6900 | 0.7474 |
| cg27630820 | 0.0008 | *PIWIL1* | 130822294 | chr12:130821453-130824831 | 0.6713 | 0.6360 | 0.6251 | 0.6946 |
| cg19424457 | 0.0009 | *PIWIL1* | 130822308 | chr12:130821453-130824831 | 0.5716 | 0.5491 | 0.5346 | 0.6070 |
| cg11931211 | 0.0013 | *PIWIL1* | 130822361 | chr12:130821453-130824831 | 0.6194 | 0.5897 | 0.5767 | 0.6476 |
| cg24838063 | 0.0054 | *PIWIL1* | 130822603 | chr12:130821453-130824831 | 0.7889 | 0.7399 | 0.7537 | 0.7976 |
| cg15166039 | 0.0010 |  | 160023626 | chr6:160023581-160024144 | 0.4976 | 0.4876 | 0.4553 | 0.5137 |
| cg26873880 | 0.0009 |  | 160023689 | chr6:160023581-160024144 | 0.7513 | 0.7472 | 0.7124 | 0.7718 |
| cg10672567 | 0.0007 |  | 160023927 | chr6:160023581-160024144 | 0.5985 | 0.5813 | 0.5332 | 0.6202 |
| cg27247736 | 0.0174 | *PNLDC1* | 160241105 | chr6:160241105-160241556 | 0.5153 | 0.4979 | 0.4522 | 0.4960 |
| cg17481235 | 0.0124 | *PNLDC1* | 160241360 | chr6:160241105-160241556 | 0.3015 | 0.2743 | 0.2457 | 0.2781 |
| cg21457401 | 0.0129 | *PNLDC1* | 160241519 | chr6:160241105-160241556 | 0.4939 | 0.4629 | 0.4310 | 0.4748 |
| cg19626725 | 0.0004 | *RUFY1* | 178986131 | chr5:178986131-178987429 | 0.4917 | 0.5516 | 0.5330 | 0.5189 |
| cg00080972 | 0.0002 | *RUFY1* | 178986291 | chr5:178986131-178987429 | 0.1525 | 0.2050 | 0.1819 | 0.1744 |
| cg25658438 | 0.0002 | *RUFY1* | 178986372 | chr5:178986131-178987429 | 0.1999 | 0.2678 | 0.2289 | 0.2292 |
| cg21226059 | 0.0002 | *RUFY1* | 178986404 | chr5:178986131-178987429 | 0.1711 | 0.2231 | 0.1957 | 0.1950 |
| cg14820908 | 0.0002 | *RUFY1* | 178986412 | chr5:178986131-178987429 | 0.1586 | 0.2103 | 0.1798 | 0.1793 |
| cg06118287 | 0.0002 | *RUFY1* | 178986559 | chr5:178986131-178987429 | 0.1840 | 0.2366 | 0.2021 | 0.2002 |
| cg02136620 | 0.0002 | *RUFY1* | 178986620 | chr5:178986131-178987429 | 0.2448 | 0.3253 | 0.2804 | 0.2814 |
| cg01955137 | 0.0002 | *RUFY1* | 178986625 | chr5:178986131-178987429 | 0.1722 | 0.2282 | 0.1995 | 0.1913 |
| cg08058472 | 0.0002 | *RUFY1* | 178986638 | chr5:178986131-178987429 | 0.1303 | 0.1820 | 0.1514 | 0.1477 |
| cg20199595 | 0.0002 | *RUFY1* | 178986677 | chr5:178986131-178987429 | 0.1477 | 0.2007 | 0.1686 | 0.1655 |
| cg09060608 | 0.0002 | *RUFY1* | 178986726 | chr5:178986131-178987429 | 0.1617 | 0.2154 | 0.1838 | 0.1791 |
| cg05457628 | 0.0002 | *RUFY1* | 178986728 | chr5:178986131-178987429 | 0.1475 | 0.1982 | 0.1746 | 0.1676 |
| cg26516362 | 0.0004 | *RUFY1* | 178986906 | chr5:178986131-178987429 | 0.2256 | 0.2769 | 0.2435 | 0.2418 |

Ce: control participants with low ELA level, CE: control participants with high ELA level, Se: participants suffering from SAD with low ELA levels, SE: participants suffering from SAD with high ELA levels.


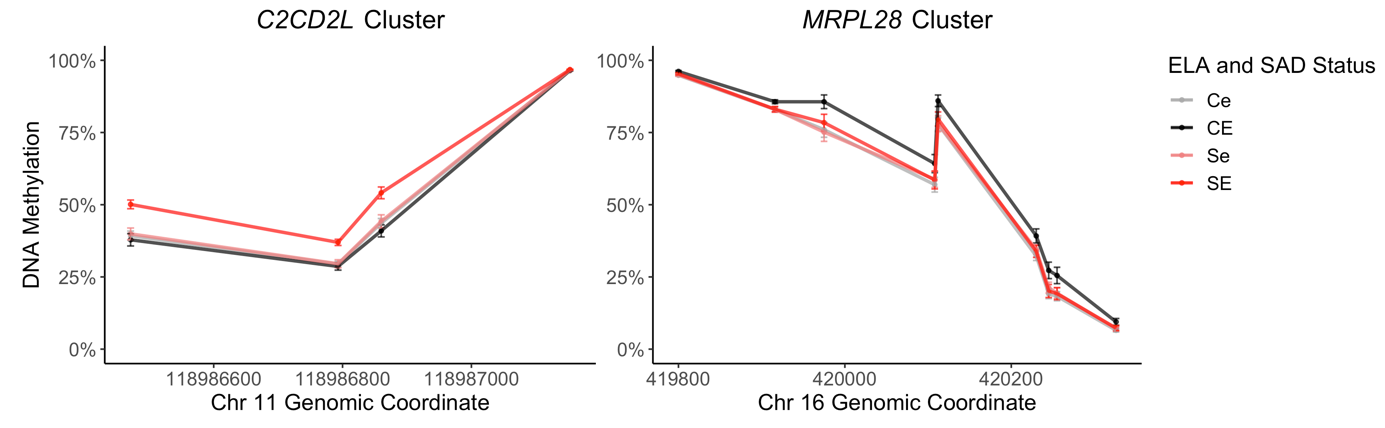


**Supplementary Figure S4.** **DMRs with respect to the interaction of SAD and ELA.** Group mean DNAm ± standard error within the top two DMRs associated with the interaction of SAD and ELA (n=143). Ce: control participants with low ELA level, CE: control participants with high ELA level, Se: participants suffering from SAD with low ELA levels, SE: participants suffering from SAD with high ELA levels.


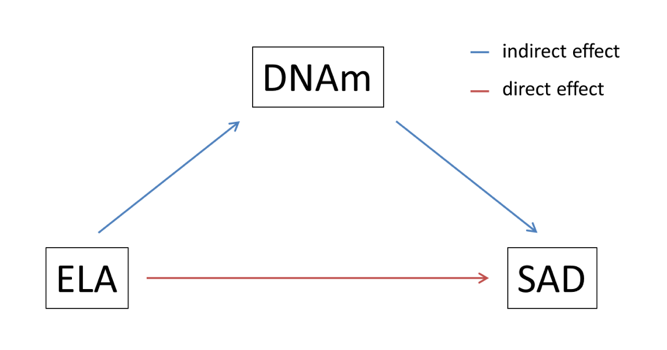


**Supplementary Figure S5. Mediation model.** Visualization of direct and indirect effects (mediated by DNAm) of ELA on SAD.
